# Spotted Fever Group and Typhus Group Rickettsioses in Humans, South Korea

**DOI:** 10.3201/eid1102.040603

**Published:** 2005-02

**Authors:** Yeon-Joo Choi, Won-Jong Jang, Jong-Hyun Kim, Ji-Sun Ryu, Seung-Hyun Lee, Kyung-Hee Park, Hyung-Suk Paik, Young-Sang Koh, Myung-Sik Choi, Ik-Sang Kim

**Affiliations:** * Konkuk University, Choongbuk, Republic of Korea;; † Pusan National University, Pusan, Republic of Korea;; ‡ Cheju National University College of Medicine, Jeju, Republic of Korea;; § Seoul National University College of Medicine and Institute of Endemic Disease, Seoul, Republic of Korea

**Keywords:** molecular detection, nested PCR, RFLP, Spotted fever group rickettsiosis, Typhus group rickettsiosis, research

## Abstract

The presence of the nucleic acid of the spotted fever group (SPG) and typhus group (TG) rickettsiae was investigated in 200 serum specimens seropositive for SFG rickettsiae by multiplex-nested polymerase chain reaction with primers derived from the rickettsial outer membrane protein B gene. The DNA of SFG, TG, or both rickettsiae was amplified in the 24 serum specimens, and sequence analysis showed *Rickettsia conorii*, *R. japonica*, and *R. felis* in the specimens. *R. conorii* and *R. typhi* were found in 7 serum specimens, which indicated the possibility of dual infection in these patients. These findings suggest that several kinds of rickettsial diseases, including boutonneuse fever, rickettsialpox, *R. felis* infection, and Japanese spotted fever, as well as scrub typhus and murine typhus, are occurring in Korea.

## Abstract 2

Multiplex-nested PCR and sequencing analysis indicated rickettsialike agents in serum specimens from febrile patients.

Human rickettsioses, known to occur in Korea, include mainly scrub typhus, murine typhus, and epidemic typhus. Scrub typhus, caused by *Orientia tsutsugamushi*, a major rickettsial disease in Korea, is transmitted through the bites of mite larvae. An earlier study by Choi and colleagues reported that 34.3% of febrile hospital patients in autumn were seropositive for the disease (*1*). *Rickettsia typhi*, transmitted by the fleas of various rodents, causes murine typhus, which is a milder form of typhus than human typhus (*2*). The first patient with murine typhus in Korea was reported in 1959. Two cases of murine typhus confirmed by culture were reported since 1988 (*3**,**4*), and now >200 cases of murine typhus are presumed to occur annually in South Korea. Epidemic typhus is caused by *R. prowazekii* and is transmitted by the body louse (*5*). The disease is fatal in 10% to 30% of patients, depending on underlying diseases and the nutritional state of the host (*2*). The disease appeared after the end of the Korean War. Since 1951, however, no other cases have been reported in Korea (*6*).

Spotted fever group (SFG) rickettsioses are associated with arthropods, such as ticks, mites, and fleas (*2*). SFG comprises several divergent lineages: the *R. rickettsii* group, *R. japonica*, *R. montana*, the *R. massiliae* group, *R. helvetica*, *R. felis*, and the *R. akari* group (*2*). Recently, the nucleic acids of *R. japonica* and *R. rickettsii* were found in *Haemaphysalis longicornis* in Korea (*7*). A previous seroepidemiologic study demonstrated that SFG rickettsioses were highly likely in Korea (*8*). No clinical human case of SFG rickettsioses, however, has been reported in Korea until now.

In this study, to check whether SFG rickettsioses were present in humans, serum specimens from patients with acute febrile disease were studied by using molecular sequence–based identification techniques. We report the presence of the *rompB* gene of SFG rickettsiae, similar to *R. akari*, *R. conorii*, *R. japonica*, and *R. felis*, in serum specimens from Korean patients with acute febrile disease. The nucleic acids of both *R. conorii* and *R. typhi* were found to coexist in 7 serum specimens. This study presents the first molecular evidence of SFG rickettsioses in humans.

## Materials and Methods

### Rickettsial Strains

The following strains were obtained from the American Type Culture Collection (ATCC; Manassas, VA, USA): *R. typhi* Wilmington (VR-144), *R. prowazekii* Breinl (VR-142), *R. akari* MK (VR-148), *R. japonica* YH (VR-1363), *R. conorii* Indian Tick Typhus (VR-597), and *R. sibirica* 246 (VR-151). These rickettsial agents were propagated in Vero (CRL-1586) or L929 (CCL-1) cell monolayers.

### Serum Samples and Serologic Testing

The serum specimens analyzed in this study were obtained from South Korean patients with acute febrile illness from 1993 to 1999. The specimens were submitted to the Institute of Endemic Disease at Seoul National University's Medical Research Center for laboratory diagnosis for scrub typhus, leptospirosis, and hemorrhagic fever with renal syndrome caused by hantavirus. Some of the serum specimens were used for the nucleic acid detection study of SFG rickettsial agents. The rationale for selecting the samples for polymerase chain reaction (PCR) analysis included the presence of immunoglobulin (Ig) M antibodies with titers from 1:40 to 1:160 against any of the tested antigens in the samples. Serologic testing was performed by indirect immunofluorescence assay (IFA) with a panel of 4 SFG rickettsial antigens, *R. japonica*, *R. akari*, *R. conorii*, and *R. sibirica*, as previously described (*8*).

### Oligonucleotide Primers

The oligonucleotide primers used for priming the PCRs are shown in Table 1. The primers were developed on the basis of the *rompB* gene sequences of *R. conorii* strain Seven (GenBank accession no. AF123721), and the citrate synthase (*gltA*) gene sequence of *R. prowazekii* (GenBank accession no. M17149) was synthesized. The selection of the primers was based on the "primer 3" program (http://www-genome.wi.mit.edu/cgi-bin/primer/primer3_www.cgi/), to obtain the optimal melting temperature and GC content and to avoid hairpin loop structures. The selected sequences were analyzed through the BLAST program (http://www.ncbi.nlm.nih.gov/BLAST/).

**Table 1:** Oligonucleotide primers for amplification of partial rickettsial genes*

Primer	Target rickettsia group	Gene	Position	Nucleotide sequence (5´–3´)
rompB OF	SFG and TG	*rompB†*	3,620–3,643	GTAACCGGAAGTAATCGTTTCGTAA
rompB OR	SFG and TG	*rompB*	4,131–4,109	GCTTTATAACCAGCTAAACCACC
rompB SFG IF	SFG	*rompB*	3,652–3,674	GTTTAATACGTGCTGCTAACCAA
rompB SFG/TG IR	SFG and TG	*rompB*	4,077–4,057	GGTTTGGCCCATATACCATAAG
rompB TG IF	TG	*rompB*	3,828–3,850	AAGATCCTTCTGATGTTGCAACA
RpCS.877p§	SFG and TG	*gltA‡*	877–895	GGGGGCCTGCTCACGGCGG
RpCS.1,258n§	SFG and TG	*gltA*	1,258–1,237	ATTGCAAAAAGTACAGTGAACA
RpCS.896p	SFG and TG	*gltA*	896–915	GGCTAATGAAGCAGTGATAA
RpCS.1,233n	SFG and TG	*gltA*	1,233–1,215	GCGACGGTATACCCATAGC

*SFG, spotted fever group; TG, typhus group; OR, outer reverse primer; OF, outer forward primer. †Oligonucleotide primer sequences derived from *Rickettsia conorii* genes (accession no. AF123721). ‡Oligonucleotide primer sequences derived from *R. prowazekii* genes (accession no. M17149). §Primer sequences derived from (*9*).

### Detection of *rompB* Gene in Human Sera

DNA for PCR analysis was extracted from 200 mL of serum samples by using QIAamp Blood Mini Kit (Qiagen GmbH, Hilden, Germany) according to the manufacturer's instructions. SFG and typhus group (TG) rickettsia *rompB* gene in human sera were detected with multiplex nested PCR. The primary amplification of the specimen was performed in a final reaction volume of 50 µL. The reaction mixture contained 5 µL of prepared DNA sample, 20 pmol of *rompB* outer forward primer (OF) and outer reverse primer (OR), 200 µM of deoxynucleoside triphosphate mixture (dNTP, Takara, Otsu, Japan), 1 x PCR buffer, 1.25 U Taq polymerase (Takara EX Taq, Takara), and distilled water. First, PCR reactions were incubated at 95°C for 5 min, subjected to 35 cycles of 95°C for 15 s, 54°C for 15 s, and 72°C for 30 s, and final extension at 72°C for 3 min in a GeneAmp PCR system 9600 (Perkin-Elmer Applied Biosystems, Foster City, CA, USA). After this, 2 µL of the amplified product was again amplified in a nested fashion with inner primer sets (rompB SFG IF, rompB SFG/TG IR, and rompB TG IF). The nested PCR reaction mixture contained 10 pmol of each primer in a PCR premixture tube (AccuPower PCR PreMix, Bioneer Corp., Daejon, Korea) that contained 1 U of Taq DNA polymerase, 250 mmol/L each of dNTP, 50 mmol/L of Tris-HCl (pH 8.3), 40 mmol/L of KCl, 1.5 mmol/L of MgCl_2_, and gel loading dye. The volume was then adjusted to 20 µL with distilled water. Nested PCR reactions were incubated at 95°C for 5 min, subjected to 35 cycles of 95°C for 15 s, 56°C for 15 s, and 72°C for 30 s, and final extension at 72°C for 3 min. PCR amplification of the *gltA* gene of SFG and TG rickettsiae was performed by using the oligonucleotide pairs RpCS.877p and RpCS.1,258n for the primary PCR amplification and RpCS.896p and RpCS.1,233n for the secondary amplification. The primary PCR cycling condition consisted of incubation at 95°C for 5 min, then 35 cycles each of 15 s at 95°C, 15 s at 54°C, and 30 s at 72°C, followed by a final extension cycle of 3 min at 72°C. The nested PCR cycling condition consisted of incubation at 95°C for 5 min, then 35 cycles each of 15 s at 95°C, 15 s at 54°C, and 30 s at 72°C, followed by a final extension cycle of 3 min at 72°C. To avoid cross-contamination, 3 separate rooms with entirely separate equipment and solutions were used. Thus, the handling and treatment of samples and the addition of a template, the handling of DNA-free PCR reagents, and the post-PCR work were strictly separated. Aerosol-resistant tips (Axigen Scientific, Inc., Union City, CA, USA) were used for the handling of all reagents in the PCR study. The amplification products were visualized by electrophoresis on a 1.5% agarose gel stained with ethidium bromide (0.5 µg/mL) and using 1 x TAE migration buffer (pH 8.0; 40 mmol/L Tris-acetate, 1 mmol/L EDTA).

### Restriction Fragment Length Polymorphism (RFLP) Analysis

The PCR products were purified by using an AccuPrep PCR purification kit (Bioneer Corp.), according to the manufacturer's instructions. Restriction endonuclease digestions were performed with 10 µL of amplified products by using *Alu*I (New England Biolabs, Beverly, MA, USA). The digested DNA was resolved by electrophoresis through a 10% polyacrylamide gel at 100 V for 4 h in a 1 x TBE buffer (pH 8.0; 90 mmol/L Tris-borate, 2 mmol/L EDTA), and was visualized after staining with ethidium bromide.

### Cloning, Sequencing, and Analysis of Nucleotide

All positive PCR products were cloned by using pGEM-T Easy Vector System I (Promega). Verifying whether the clones contained inserts was accomplished by digestion of plasmid DNA with EcoRI (New England Biolabs) and separation in 1.5% agarose gels. Plasmids containing DNA inserts were sequenced for both strands by using Big Dye Terminator Sequence Kit and ABI Prism 377 Automated DNA Sequencer (Perkin-Elmer Applied Biosystems), according to the manufacturer's protocol. The obtained sequences, except for the primer regions, were aligned with the corresponding sequences of other rickettsiae deposited in the GenBank database to identify known sequences with a high degree of similarity using multisequence alignment programs, the Phydit software (*10*), and the MegAlign software package (Windows version 3.12e; DNASTAR, DYNASTAR Inc., Madison, WI, USA). Phylogenetic trees were generated by using the neighbor-joining algorithms and the Jukes and Cantor matrix. Bootstrap analysis was performed to investigate the stability of the trees obtained through the neighbor-joining method. The percentages of similarity were determined using the FASTA network service (European Bioinformatics Institute Fasta Service; available from http://www.ebi.ac.uk/fasta).

### Nucleotide Sequence Accession Numbers Used

GenBank accession numbers of the *rompB* gene sequences used for sequence comparisons are AB003681 for *R. japonica*, AF123705 for *R. aeschlimannii*, AF123706 for *R. africae*, AF123707 for *R. akari*, AF123708 for Astrakhan rickettsia strain A-167, AF123709 for *R. australis*, AF123711 for *R. honei* strain RB, AF123712 for Israeli tick typhus rickettsia, AF123714 for *R. massiliae*, AF123715 *R. mongolotimonae*, AF123716 for *R. montanensis*, AF123717 for *R. parkeri*, AF123719 for *R. rhipicephali*, AF123721 for *R. conorii* strain Seven, AF123722 for *R. sibirica*, AF123723 for *R. slovaca*, AF123725 for *R. helvetica*, AF182279 for *R. felis*, AF211820 for *R. prowazekii* strain Florida, AF211821 for *R. prowazekii* strain Virginia, AF123718 for *R. prowazekii*, AF161079 for *R. prowazekii*, AF479763 for *R. amblyommii* strain WB-8-2 rompB pseudogene, AY260451 for *R. heilongjiangensis*, AY260452 for *R. hulinensis*, L04661 for *R. typhi* crystalline surface layer protein (slpT) gene, and X16353 for *R. rickettsii*. The GenBank accession number of the *gltA* gene sequence used for developing primers is M17149 for *R. prowazekii*.

## Results

### Multiplex Nested PCR Amplification of *rompB* Gene

**Figure 1:**
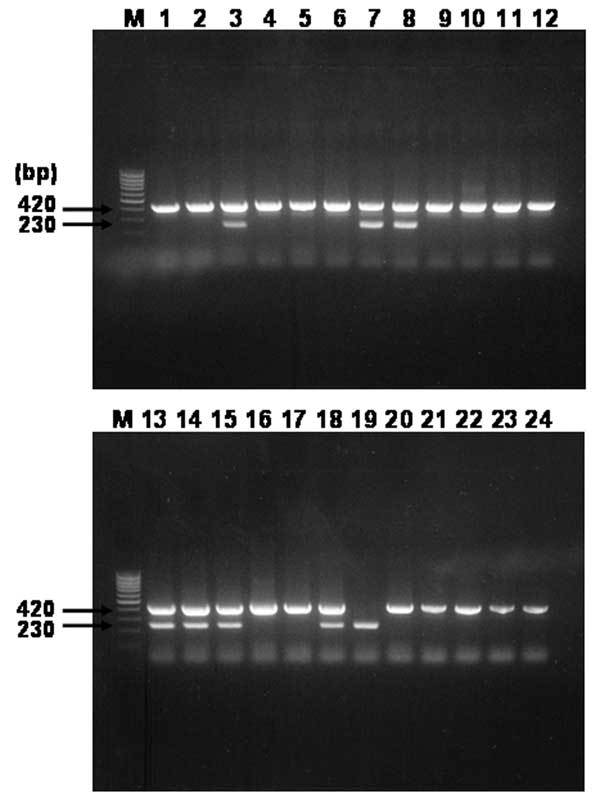
Agarose gel electrophoresis analysis on 1.5% agarose gel of DNA sequences amplified by multiplex-nested polymerase chain reaction (PCR) assay by using outer and inner primer sets targeted *rompB* gene and template DNAs from serum samples. Lanes: M, size marker DNA (100-bp DNA ladder); 1–24, each number of amplified H products. The number on the left indicates the molecular size (in base pairs) of the amplified PCR products.

Nested PCR assay, with primer pairs rompB OF and rompB OR in primary reactions and rompB SFG IF, rompB SFG/TG IR, and rompB TG IF in multiplex-nested reactions, was performed to identify the unknown rickettsial agents in the seropositive serum specimens and to differentiate between SFG and TG rickettsiae in terms of size. When the primers previously mentioned were used, the nested PCR assay generated ≈420 bp for SFG rickettsiae and about 230 bp for TG rickettsiae. The negative controls consistently failed to yield detectable PCR products, whereas the positive controls always gave the expected PCR products. Overall, 200 serum specimens from febrile patients from all areas of South Korea were tested. After the nested PCR was performed, the expected *rompB* gene products were obtained from 24 seropositive serum samples. Figure 1 shows the result of electrophoresis of 24 PCR-amplified samples. Of the 24 amplified products, 16 showed the electrophoretic pattern of 1 DNA band of ≈420 bp, which corresponded to SFG. The amplified size of only 1 sample was ≈230 bp for TG. The 7 other amplified products showed an electrophoretic pattern of 2 bands of ≈420 bp for SFG and 230 bp for TG. Therefore, the 23 amplified products corresponding to SFG rickettsial agents were named H1 product, while the 8 products corresponding to TG were named H2 product. The H1 products included H1 to H24 (except H19), while the H2 products were H3-2, H7-2, H8-2, H13-2, H14-2, H15-2, H18-2, and H19 (Figure 1).

### RFLP Analysis and Sequencing Analysis

**Figure 2:**
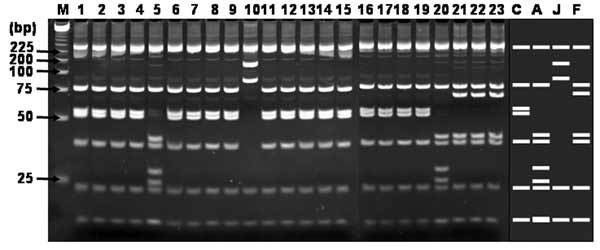
Restriction fragment length polymorphism analysis of H1 products amplified with multiplex-nested primer set from seropositive sera. Ethidium bromide–stained polyacrylamide gels of *Alu*I restriction endonuclease digestion of ≈420 bp rickettsial DNA amplified by using the nested primer H set WJ77/80 in the primary reactions and WJ79/83/78 in the nested reactions. Lanes: M, size marker DNA (25-bp DNA ladder); 1–18: H1–H18; 19–23: H20–24; C, *Rickettsia conorii*; A, *R. akari*; J, *R. japonica*; F, *R. felis*. J–S; predicted fragments after digestion. The number on the left indicates the molecular size (in base pairs) of restriction fragments.

**Figure 3:**
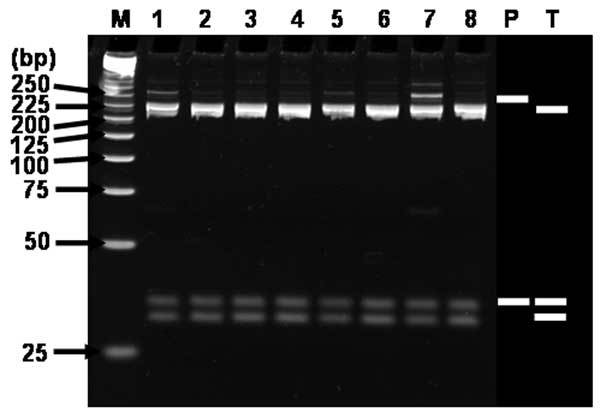
Restriction fragment length polymorphism analysis of H2-products amplified with multiplex-nested primer set from seropositive sera. Ethidium bromide–stained polyacrylamide gels of *Alu*I restriction endonuclease digestion of ≈230-bp rickettsial DNA amplified by using the nested primer H set WJ77/80 in the primary reactions and WJ79/83/78 in the nested reactions. Lanes: M, size marker DNA (25-bp DNA ladder); 1, H3-2; 2, H7-2; 3, H8-2; 4, H13-2; 5, H14-2; 6, H15-2; 7, H18-2; 8, H19; P, *Rickettsia prowazekii*; T, *R. typhi*. P and T; predicted fragments after digestion. The number on the left indicates the molecular size (in base pairs) of restriction fragments.

RFLP analysis of the 23 H1 products corresponding to SFG rickettsial agents using *Alu*I demonstrated that the restriction patterns of 17 H1 products were identical with that of *R. conorii*, 2 with that of *R. akari*, 1 with that of *R. japonica*, and 3 with that of *R. felis* (Figure 2). RFLP analysis of the 8 H2 products corresponding to TG rickettsial agents by using *Alu*I showed that the restriction patterns of all the H2 products were identical with that of *R. typhi* (Figure 3).

### Sequencing Analysis

To identify the SFG and TG rickettsiae detected in human serum specimens, nucleotide sequences of the PCR-amplified products were determined and compared with partial *rompB* gene sequences of various rickettsial agents obtained from the GenBank database. Table 2 shows the similarity between the partial *rompB* gene sequences of various rickettsial agents and 6 of the sequenced H1 products (clones H1, H3, H5, H10, H20, and H22). Clones H1, H3, and H20 showed 100%, 99.72%, and 98.87% degrees of similarity to *R. conorii*, respectively. Clone H10 showed 100% similarity to *R. japonica*, and clone H5 showed 100% similarity to *R. akari*. In particular, clone H22 showed 99.44% similarity to *R. felis*. All the compared H1 products showed low levels of similarity (70.90%–74.01%) to the TG species. The clones that clustered partially with the *rompB* gene of *R. conorii* were differentiated in 3 groups by their levels of similarity: group 1 (12 H1 products with 100% similarity), group 2 (4 H1 products with 99.72% similarity), and group 3 (1 H1 product with 98.87% similarity). Clones H22, H23, and H24 clustered as the *R. felis* group. Table 3 shows the similarity between the partial *rompB* gene sequences of various rickettsial species and H2 product sequences. All H2 products showed low levels of similarity (67.05%–69.94%) to SFG rickettsial species, such as *R. sibirica*, *R. akari*, *R. conorii*, *R. felis*, and *R. japonica*. They also showed high levels of similarity (93.64%–100%) to TG rickettsial species, such as *R. prowazekii* and *R. typhi*. The H2 products' levels of similarity to *R. typhi* ranged from 99.42% to 100%. A neighbor-joining analysis based on partial *rompB* gene sequences demonstrated that 17 H1 products formed a cluster with *R. conorii*, 2 with *R. akari*, 1 with *R. japonica*, and 3 with *R. felis* (data not shown). The analysis of the 8 H2 product sequences showed that the sequences of all H2 products formed a cluster with *R. typhi* and were separated from the SFG rickettsial strains (data not shown).

**Table 2:** Similarity matrix between partial *rompB* gene sequence of various rickettsial strains and nested polymerase chain reaction (H1 products)

	1*	2	3	4	5	6	7	H1†	H3	H5	H10	H20	H22
1*	–												
2	93.79	–											
3	96.05	94.92	–										
4	91.81	96.89	92.94	–									
5	92.94	98.59	94.07	96.61	–								
6	74.01	73.16	74.29	72.32	73.45	–							
7	72.03	70.9	72.32	70.34	71.19	93.22	–						
H1†	93.79‡	100	94.92	96.89	98.59	73.16	70.9	–					
H3	93.5	99.72	94.63	96.61	98.31	72.88	70.62	99.72	–				
H5	100	93.79	96.05	91.81	92.94	74.01	72.03	93.79	93.5	–			
H10	91.81	96.89	92.94	100	96.61	72.32	70.34	96.89	96.61	91.81	–		
H20	92.66	98.87	93.79	96.33	97.46	72.6	70.34	98.87	98.59	92.66	96.33	–	
H22	95.76	94.63	99.44	92.66	93.79	74.01	72.03	94.63	94.35	95.76	92.66	93.5	–

*1, partial *rompB* of *Rickettsia akari* (AF123707), 2; *R. conorii* (AF123721); 3, *R. felis* (AF182279); 4, *R. japonica* (AB003681); 5, *R. sibirica* (AF12322); 6, *R. prowazekii* (AF211820); 7, *R. typhi* (L04661). †H, H1 products amplified from patient sera. ‡The similarity values (%) on the lower left are the levels of similarity between partial *rompB* gene sequences.

**Table 3:** Similarity matrix between partial *rompB* gene sequence of various rickettsial strains and nested polymerase chain reaction (H2 products)

	1*	2	3	4	5	6	7	8	9	10	H8-2†	H13-2	H14-2	H15-2	H18-2	H19	H3-2	H7-2
1*	–																	
2	92.49	–																
3	98.84	93.64	–															
4	94.8	95.38	95.95	–														
5	95.38	90.17	96.53	92.49‡	–													
6	67.63	70.52	67.63	68.79	67.05	–												
7	67.63	70.52	67.63	68.79	67.05	100	–											
8	67.63	70.52	67.63	68.79	67.05	100	100	–										
9	67.63	70.52	67.63	68.79	67.05	100	100	100	–									
10	67.63	69.94	67.63	69.36	67.05	94.22	94.22	94.22	94.22	–								
H8-2†	67.63	69.94	67.63	69.36	67.05	94.22	94.22	94.22	94.22	100	–							
H13-2	67.05	69.36	67.05	68.79	66.47	93.64	93.64	93.64	93.64	99.42	99.42	–						
H14-2	67.63	69.94	67.63	69.36	67.05	94.22	94.22	94.22	94.22	100	100	99.42	–					
H15-2	67.63	69.94	67.63	69.36	67.05	94.22	94.22	94.22	94.22	100	100	99.42	100	–				
H18-2	68.21	70.52	68.21	69.94	67.63	93.64	93.64	93.64	93.64	99.42	99.42	98.84	99.42	99.42	–			
H19	67.63	69.94	67.63	69.36	67.05	94.22	94.22	94.22	94.22	100	100	99.42	100	100	99.42	–		
H3-2	67.63	69.94	67.63	69.36	67.05	94.22	94.22	94.22	94.22	100	100	99.42	100	100	99.42	100	–	
H7-2	67.63	69.94	67.63	69.36	67.05	94.22	94.22	94.22	94.22	100	100	99.42	100	100	99.42	100	100	–

*1, partial *rompB* genes of *Rickettsia sibirica* (AF12322); 2, *R. akari* (AF123707), 3; *R. conorii* (AF123721); 4, *R. felis* (AF182279); 5, *R. japonica* (AB003681); 6, *R. prowazekii* (AF211820); 7, *R. prowazekii* (AF123718); 8, *R. prowazekii* (AF161079); 9, *R. prowazekii* (AF211821); 10, *R. typhi* (L04661). †H, H2 products amplified from patient sera. ‡The similarity values (%) on the lower left are the levels of similarity between partial *rompB* gene sequences and the values (bp) on the upper right are the number of different bases out of compared total bases in partial *rompB* gene sequences.

### Nested PCR Amplification of *gltA* Gene

**Figure 4:**
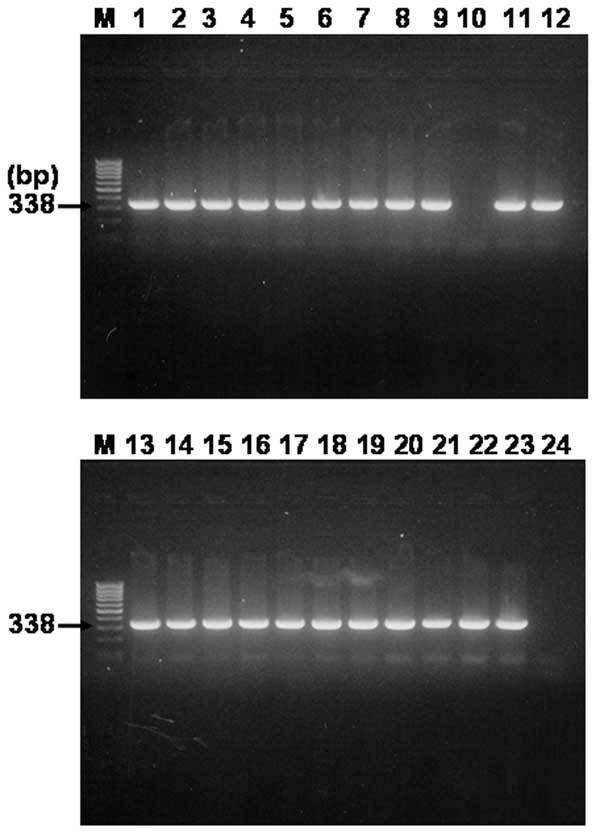
Agarose gel electrophoresis analysis on 1.5% agarose gel of DNA sequences amplified by nested polymerase chain reaction (PCR) assay using primer sets targeted partial *gltA* gene and template DNA sequences from 24 serum samples. Lanes: M, size marker DNA (100-bp DNA ladder); 1–24, each number of amplified *gltA* products. The number on the left indicates the molecular size (in base pairs) of the amplified PCR products.

The results of the multiplex nested PCR of the *rompB* gene were confirmed by a second PCR assay with specific primer pairs RpCS.877p and RpCS.1,258 in primary reactions and RpCS.896p and RpCS.1,233 in nested reactions. The primer sets generated ≈338 bp for SFG and TG rickettsiae. The expected size of the *gltA* gene fragment was generated in 22 of 24 samples that were positive for the PCR detection of the *rompB* gene (Figure 4). All positive PCR products were cloned, and their sequences were determined. Since the PCR assay using primer sets for the amplification of the *gltA* gene could not discriminate between the SFG rickettsia and TG rickettsia by size difference, the sequences of 3 clones for each PCR product were determined. The results of the sequencing analysis for *gltA*-PCR amplifications were identical to those of the analysis of the rompB-PCR product (data not shown). Seven samples that were positive for both the *rompB* genes of *R. conorii* and *R. typhi* were also positive for both of their *gltA* genes (data not shown).

## Discussion

SFG and TG rickettsial infections occur worldwide and may cause serious diseases in humans. These pathogenic bacteria are transmitted to people by arthropod vectors, such as ticks, fleas, and lice. In this study, multiplex-nested PCR was conducted to detect and identify SFG and TG rickettsial antigens in patient sera with positive results from the serosurvey. The *rompB* gene domain II region, which is a highly conserved region of *rompB*, was targeted for PCR amplification for the specific detection of SFG and TG rickettsiae. Amplified DNA sequences were analyzed by using nucleotide-sequencing methods, and RFLP analysis was used to confirm the PCR results. The results indicated the presence of several SFG rickettsiae, *R. conorii*, *R. akari*, *R. japonica*, and *R. felis*, in the serum specimens. The results were also confirmed by a second PCR with specific primer pairs for the *gltA* gene and by sequence analysis of its DNA amplicons.

For the first time, SFG rickettsiae in human serum specimens in South Korea have been reported. *R. akari* is a member of the spotted fever group rickettsiae and is a causative agent of rickettsial pox, a disease transmitted by the bite of *Allodermanyssus sanguineus*, a mite ectoparasite of the domestic mouse (*Mus muscularis*) (*2*). The disease was first described in New York City in 1946. *R. akari* was isolated from the Korean vole in 1957. The previous seroepidemiologic study conducted by the authors on 3,401 patients with febrile disease indicated that the seropositive rate was 16.24% for the rickettsial antigen through IFA. *R. conorii* is an etiologic agent of the Mediterranean spotted fever or boutonneuse fever (*2*). Our previous study indicated that the seropositive rate was 14.34% for the antigen. *R. japonica*, the causative agent of Oriental spotted fever, was first isolated from a patient with febrile, exanthematous illness in Japan in 1985 (*2*). The disease is now endemic in the southwestern part of Japan, where >100 cases have been described (*2*). Previous studies showed the presence of nucleic acids of *R. japonica* and *R. rickettsii* in *H. longicornis* by PCR. Our seroepidemiologic study demonstrated that the seropositive rate was 19.9%. Although no clinical human case of SFG rickettsioses has been reported in Korea until now, this study's findings strongly suggest the prevalence of SFG rickettsiosis in Korea.

*R. felis* is an emerging pathogen responsible for fleaborne spotted fever and had been considered a member of the TG rickettsiae based on its reactivity with anti–*R. typhi* antibodies. A genetic analysis of the 16S rRNA, citrate synthase, *rompA*, and *rompB* genes, however, placed *R. felis* as a member of SFG. *R. felis* has been reported in various countries, including the United States, Mexico, Brazil, Germany, and France (*11**,**12*). In Asia, the first case of *R. felis* infection was reported in 2003 (*13*). *R. typhi* was also among those detected in the SFG rickettsiae in the febrile disease patients' sera. Fleas are also found to be vectors for *R. typhi* (*2*). Of major importance to the epidemiology of the rickettsioses caused by *R. typhi* and *R. felis* is the maintenance of both rickettsial agents in their hosts by transovarial transmission, and the fact that neither organism is lethal for fleas (*14*).

Finally, we report the presence of both *R. conorii* and *R. typhi* in serum from Korean patients. Sera from patients with SFG rickettsiosis have been reported to react with TG rickettsiae by using serologic analysis methods (*15*). The serum specimens from patients with TG rickettsiosis were also demonstrated to contain cross-reactive antibodies against SFG rickettsiae (*15**,**16*). In a previous study, approximately one third of specimens seropositive for antibodies against SFG rickettsiae had antibodies against TG rickettsiae (unpub. data). Therefore, the multiplex-nested PCR was designed to detect and differentiate SFG rickettsial agents from TG rickettsial agents in the patient serum specimens with positive results from the serosurvey with SFG rickettsial antigens. SFG rickettsiae and TG rickettsiae were differentiated in terms of the size of amplified products. PCR results also confirmed the RFLP and sequencing analysis. In sera taken from 7 patients, both SFG and TG rickettsial antigens were detected, which indicated dual infection. Previously, a case of dual infection with *Ehrlichia chaffeensis* and an SPG rickettsia was reported in a human patient. Cases of dual infection with *Bartonella clarridgeiae* and *B. henselae* in cats have also been reported, as well as infection with the 2 different genotypes of *B. henselae* (*17**,**18*). A recent report suggested that coinfection of *R. felis* with either *B. clarridgeiae* or *B. quintana* in fleas may cause dual infection in a human that comes in contact with flea feces (*14*). These reports support this study's findings regarding the dual infection of SFG and TG rickettsiae in 7 patients. The differences between *R. conorii* and *R. typhi* vectors, however, still cannot be explained, and further studies are needed.

In conclusion, this study confirmed, by using PCR-based amplification methods, that several SFG rickettsiae, *R. conorii*, *R. akari*, *R. japonica*, and *R. felis*, existed in the sera of Korean patients with febrile episodes. Our findings indicate that SFG rickettsiae, including *R. felis*, should be used in serologic tests on Korean patients suspected of having rickettsiosis. TG rickettsiae existed in 8 patients, and 7 of them were also infected with *R. conorii*. The evidence of double infection is expected to help describe the cross-reactivity between the patient sera of SFG rickettsioses and TG rickettsioses.

## References

[1] Choi MS, Park SK, Chang WJ, Huh MS, Kim HR, Han TH, A seroepidemiological survey on the scrub typhus in Korea, 1994. J Korean Soc Microbiol. 1994;30:593–602.

[2] Raoult D, Roux V. Rickettsioses as paradigms of new or emerging infectious diseases. Clin Microbiol Rev. 1997;10:694–719. 9336669 10.1128/cmr.10.4.694 PMC172941

[3] Kim YW, Cho MK, Min CH, Yoon CS. Isolation and characterization of *Rickettsia typhi* from patients in Korea. J Korean Soc Microbiol. 1988;23:265–75.

[4] Song JW, Baek LJ, Lee YJ, Song KJ, Han SH. Seroepidemiologic analysis of acute rebrile illness from Korea in 1996. J Korean Soc Virol. 1998;28:377–82.

[5] Gross L. How Charles Nicolle of the Pasteur Institute discovered that epidemic typhus is transmitted by lice: reminiscence from my years at the Pasteur Institute in Paris. Proc Natl Acad Sci U S A. 1996;93:10539–40. 10.1073/pnas.93.20.10539 8855211 PMC38186

[6] Chung HY. Suspected human infectious diseases in Korea; Rickettsial infections. Korean J Infect Dis. 1985;18:93–7.

[7] Lee JH, Park HS, Jung KD, Jang WJ, Koh SE, Kang SS, Identification of spotted fever group rickettsiae detected from *Haemaphysalis longicornis* in Korea. Microbiol Immunol. 2003;47:301–4. 12801068 10.1111/j.1348-0421.2003.tb03399.x

[8] Jang WJ, Kim JH, Choi YJ, Jung KD, Kim YG, Lee SH, First serologic evidence of human spotted fever group rickettsiosis in Korea. J Clin Microbiol. 2004;42:2310–3. 10.1128/JCM.42.5.2310-2313.2004 15131221 PMC404613

[9] Roux V, Rydkina E, Eremeeva M, Raoult D. Citrate synthase gene comparison, a new tool for phylogenetic analysis, and its application for the rickettsiae. Int J Syst Bacteriol. 1997;47:252–61. 10.1099/00207713-47-2-252 9103608

[10] Chun J. Computer-assisted classification and identification of actinomycetes. [Ph.D. thesis]. Newcastle Tyne, United Kingdom: University of Newcastle upon Tyne; 1995.

[11] Zavala-Velazquez JE, Ruiz-Sosa JA, Sanchez-Elias RA, Becerra-Carmona G, Walker DH. *Rickettsia felis* rickettsiosis in Yucatan. Lancet. 2000;356:1079–80. 10.1016/S0140-6736(00)02735-5 11009147

[12] Richter J, Fournier PE, Petridou J, Haussinger D, Raoult D. *Rickettsia felis* infection acquired in Europe and documented by polymerase chain reaction. Emerg Infect Dis. 2002;8:207–8. 10.3201/eid0802.010293 11897076 PMC2732449

[13] Parola P, Miller RS, McDaneiel P, Telford SR III, Rolain JM, Wongsrichanalai C, Emerging rickettsioses of the Thai-Myanmar border. Emerg Infect Dis. 2003;9:592–5. 12737744 10.3201/eid0905.020511 PMC2972759

[14] Rolain JM, Franc M, Davousr B, Raoult D. Molecular detection of pathogenic *Bartonella* and *Rickettsia* in cat fleas from France. Emerg Infect Dis. 2003;9:338–42. 12643829 10.3201/eid0903.020278 PMC2958535

[15] Hechemy KE, Raoult D, Fox J, Han Y, Elliotte LB, Rawlings J. Cross-reaction of immune sera from patients with rickettsial disease. J Med Microbiol. 1989;29:199–202. 10.1099/00222615-29-3-199 2501497

[16] Uchiyama T, Zhao L, Yan Y, Uchida T. Cross-reacttivity of *Rickettsia japonica* and *Rickettsia typhi* demonstrated by immunofluorescence and Western immunoblotting. Microbiol Immunol. 1995;39:951–7. 8789054 10.1111/j.1348-0421.1995.tb03298.x

[17] Gurfield AN, Boulouis HJ, Chomel BB, Kasten RW, Heller R, Bouillin C, Epidemiology of *Bartonella* infection in domestic cats in France. Vet Microbiol. 2001;80:185–98. 10.1016/S0378-1135(01)00304-2 11295338

[18] Abbott RC, Chomel BB, Kasten RW, Floyd-Hawkins KA, Kikuchi Y, Koehler JE, Experimental and natural infection with *Bartonella henselae* in domestic cats. Comp Immunol Microbiol Infect Dis. 1997;20:41–51. 10.1016/S0147-9571(96)00025-2 9023040

